# Normal and inverted regimes of charge transfer controlled by density of states at polymer electrodes

**DOI:** 10.1038/s41467-017-01264-2

**Published:** 2017-10-19

**Authors:** M. Rudolph, E. L. Ratcliff

**Affiliations:** Department of Materials Science and Engineering, University of Arizona, 1235 E. James E. Rogers Way, Tucson, AZ 85721 USA

## Abstract

Conductive polymer electrodes have exceptional promise for next-generation bioelectronics and energy conversion devices due to inherent mechanical flexibility, printability, biocompatibility, and low cost. Conductive polymers uniquely exhibit hybrid electronic–ionic transport properties that enable novel electrochemical device architectures, an advantage over inorganic counterparts. Yet critical structure–property relationships to control the potential-dependent rates of charge transfer at polymer/electrolyte interfaces remain poorly understood. Herein, we evaluate the kinetics of charge transfer between electrodeposited poly-(3-hexylthiophene) films and a model redox-active molecule, ferrocenedimethanol. We show that the kinetics directly follow the potential-dependent occupancy of electronic states in the polymer. The rate increases then decreases with potential (both normal and inverted kinetic regimes), a phenomenon distinct from inorganic semiconductors. This insight can be invoked to design polymer electrodes with kinetic selectivity toward redox active species and help guide synthetic approaches for the design of alternative device architectures and approaches.

## Abstract (web summary)

Charge transfer at conductive polymer/liquid interfaces critically affects the performance of organic bioelectronics and energy conversion/storage devices. Here, the authors provide a mechanistic framework to describe charge-transfer rates in the context of hybrid ionic–electronic conductivity.

## Introduction

Conductive polymers have exceptional potential for low-cost, next-generation flexible and printable devices, due to ease of processing, tunability of opto-electronic properties, and biocompatibility. These attributes, coupled with the unique hybrid electronic/ionic conduction mechanism in electrochemical systems, have enabled novel device architectures. One primary example is the organic electrochemical transistor (OECT), which utilizes ions from the electrolyte to modulate the conductivity of the transistor channel^1^. OECTs have already demonstrated higher transconductance than silicon transistors for biological activity^1^, with applications to chemical and biological sensing^2^, in vivo recording of brain activity^3^, and monitoring live cellular processes^4^. Likewise conductive polymers can be used as ion pumps to control spatial and temporal ion movement, with applications to drug delivery^5, 6^. A number of other electrochemical energy-conversion and storage devices have been realized using conductive polymers, including organic electronic ratchets^7–9^, redox-flow batteries^10, 11^, supercapacitors^12, 13^, electrochromics^14, 15^, and (photo-)electrochemical cells for catalysis and water purification^16, 17^.

**Fig. 1:**
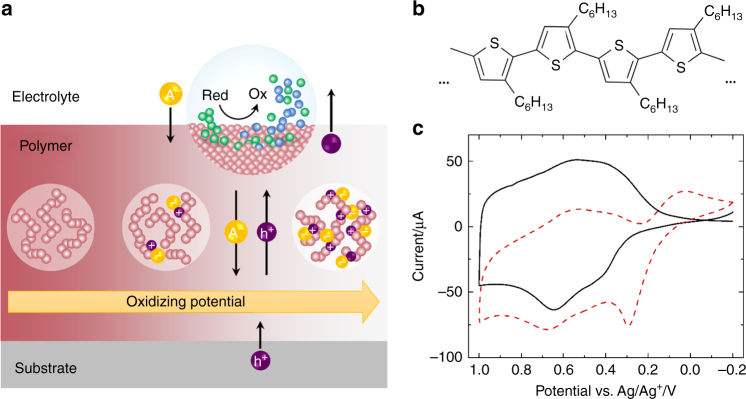
Redox reaction coupled with hybrid electronic–ionic transport at a polymer electrode. **a** Oxidation of the redox species at the polymer/electrolyte interface is enabled by potential-dependent oxidation of the polymer film coupled with intercalation of counter ions (A^−^) from the electrolyte and changes in polymer morphology (center circles). **b** Molecular structure of P3HT. **c** Cyclic voltammograms (scan rate of 50 mV s^-1^) of an e-P3HT film in the deposition bath (solid black line) and in a solution of 1 mM FcDM and 0.1 M TBAHFP in acetonitrile (dashed red line)

New polymeric and polyelectrolyte systems are rapidly emerging to simultaneously control electronic transport and volumetric doping with ions^14, 18, 19^, with an emphasis on new materials properties^20, 21^. Yet, critical structure–property relationships in electrochemical systems are still few in number relative to the synthetic knowledge found in solid-state organic electronics. In particular, key structure–property relationships to control electron transfer reactions between conductive polymer backbones and redox species within an electrolyte have received significantly less attention than the hybrid electronic–ionic conduction mechanism, despite the two processes being closely connected (Fig. 1a). Most critically, the kinetics of charge transfer directly impact the performance and efficiency of electrochemical devices, such as the change in gate voltage in the presence of a biomarker in OECT biosensors^22–24^ or over-potentials needed to produce solar fuels (e.g., H^+^ to H_2_)^16^. Selectivity to a particular redox species, in the presence of competing reactions, remains a major hurdle. What is required is an overarching understanding of charge transfer at polymer/redox electrolyte interfaces with motivating design criteria for improving the efficiency of polymer-based electrochemical devices.

In the interest of broad applicability across polymer electrochemical technologies, here we focus our studies on a model system: the well-characterized outer-sphere one-electron redox mediator 1,1′-ferrocenedimethanol^0/+^ and the widely studied organic electronic conductive polymer material poly-(3-hexylthiophene) (P3HT). This model system allows us to judge the relevance of existing electrochemical models. We demonstrate a uniting framework in which charge transfer is directly defined by the energetic overlap of the density of states (DOS) distributions in polymer and electrolyte. Both normal and inverted regimes for rates of charge transfer are observed as a function of potential; i.e., the rate is found to increase and then decrease with increasing over-potential. The inverted regime arises directly from the Gaussian distribution of electronic states characteristic of polymeric semiconductors and is not observed for inorganic semiconductor electrodes, which have square-root distributions in DOS. Demonstration of an inverted regime offers a new paradigm in multi-redox couple or multi-electron transfer mechanisms and devices, where a decreased rate of charge transfer at higher over-potentials is favorable.

## Results

### Initial assessment of electrochemical kinetics

Thin films of P3HT were electrodeposited onto indium tin oxide (ITO)-coated glass using a method introduced previously^25^. This deposition technique offers strong adhesion of the polymer film to the substrate compared to conventional deposition by spin-coating, so that films were stable throughout repetitive cycling and mechanistic interrogation. Figure 1c shows the cyclic voltammograms of electrodeposited P3HT (e-P3HT) in the presence (dashed line) and absence (solid line) of the redox mediator ferrocenedimethanol (FcDM) for the same polymer film. In the absence of a redox-active species, the difference in current in forward and reverse scans reflects the capacitive nature of the polymer: increased oxidation potential induces electronic p-doping (anodic peak at 0.65 V) coupled with the entrance of counter ions and solvent molecules into the polymeric structure to maintain charge neutrality^26, 27^. De-doping of the polymer during the reverse scan was observed as a cathodic current peak at 0.55 V.

In the presence of the redox mediator, current maxima associated with FcDM oxidation and re-reduction are observed at 0.2 V and ca. 0.1 V vs. Ag/Ag^+^, respectively. The broader shape and smaller area under the reduction feature relative to the oxidation feature indicates that electro-oxidation of FcDM by e-P3HT is generally irreversible, despite the relatively slow scan rate (50 mV s^−1^)^28^. This result serves as a first-order prediction for design criteria to control kinetic selectivity to redox-active molecules in solution; e-P3HT films are kinetically selective for FcDM oxidation.

Charge transfer at polymer/electrolyte interfaces is often evaluated using the empirical Butler–Volmer approximation to quantify a standard rate constant of electron transfer (*k* ^0^). Bobacka et al.^29^ estimated *k* ^0^ = 0.7 × 10^−3^ cm s^−1^ for the redox reaction of ferrocene at poly(3-octylthiophene) electrodes. Mandic and Duic obtained *k* ^0^ = 3.1 × 10^−3^ cm s^−1^ for the oxidation/reduction of Fe^2+/3+^ at 22-nm thick polyaniline films^30^. Following a similar methodology^31^ yields a value of *k* ^0^ ≈ 7 × 10^−4^ cm s^−1^ for the oxidation/reduction of FcDM at e-P3HT for the data in Fig. 1c, approximately 1 order of magnitude lower than a metal electrode (see Supplementary Note 2 and Supplementary Fig. 1 for full analysis and comparison).

### Role of DOS in charge transfer

In each of these analyses, one assumes metal-like behavior of the polymer electrode and introduces, a priori, an assumption that *k* ^0^ is potential-independent. Such an approach is not valid for polymer/electrolyte systems, which have significantly lower DOS than metals. We hypothesize that the electrochemically irreversible reaction observed in Fig. 1c is ascribed to a negligible energetic overlap of occupied electronic states in the polymer with unoccupied states in the electrolyte. The irreversible nature of the redox reaction provides a unique opportunity to investigate the mechanism of charge transfer, as one can consider only the forward oxidation reaction. A formal description of the total current density *J* is then approximately equal to the anodic current density *J* ^+^ based on the Marcus–Gerischer model:^32, 33^

1 $${J \approx {J^ + } \kern-1pt=\kern-1pt e{k^{\rm{t}}}{c^{{\rm{red}}}}\int_{E}^{} {\rm{DOS}}\left( E \right) \cdot \left( {1 - F\left( {E,{E_{\rm{f}}}} \right)} \right) \cdot {\rm{exp}}\left( {\frac{{ - {{\left( {E - e{E^0} - \lambda } \right)}^2}}}{{4\lambda kT}}} \right){\mathrm{d}}E,}$$

where *k* ^t^ is a time constant (in units of cm^4^ s^−1^) usually controlled by the probability of electronic tunneling, *c* ^red^ is the bulk concentration of the reduced redox species (FcDM), DOS(*E*) is the density of states distribution in the electrode, *F*(*E*, *E*_f_) is the Fermi-Dirac distribution function, *E*_f_ is the Fermi level in the electrode, and the exponential term contains the standard potential *E* ^0^ and reorganization energy *λ* of the redox electrolyte, the electron charge *e*, the Boltzmann constant *k*, and the temperature *T*.

For polymer electrodes, occupancy of the DOS changes as a function of applied potential due to electrochemical doping or de-doping combined with a structural change due to intercalation of ions. Polymer electrodes in electrolyte at steady state do not experience band bending, but rather, the position of the electronic energy levels is expected to remain homogeneous throughout the film for each potential^34, 35^. The ability to store holes through a Faradaic mechanism (i.e., the chemical capacitance *C*_µ_) is reflected in the current density *J* measured in a cyclic voltammetric experiment through *J* = *C*_µ_ × *νA* ^−1^ (where *ν* is the potential scan rate, *A* is the geometric electrode area, and *C*_µ_ is in units of *F*)^34^. Since *C*_µ_ is directly related to the DOS of the polymer, the latter can be estimated via the relationship:

2 $$\mathrm{DOS} = \frac{{{C}}}{{{e^2}\mathrm{d}A}} = \frac{J}{{{e^2}\mathrm{d}\nu }},$$

where *d* is the polymer film thickness. We stress that the thus-obtained DOS should not be considered strictly electronic, as comparable to the DOS in the solid state, but rather as an effective DOS that includes the influence of ion intercalation. A more detailed discussion is provided in Supplementary Note 1.

**Fig. 2:**
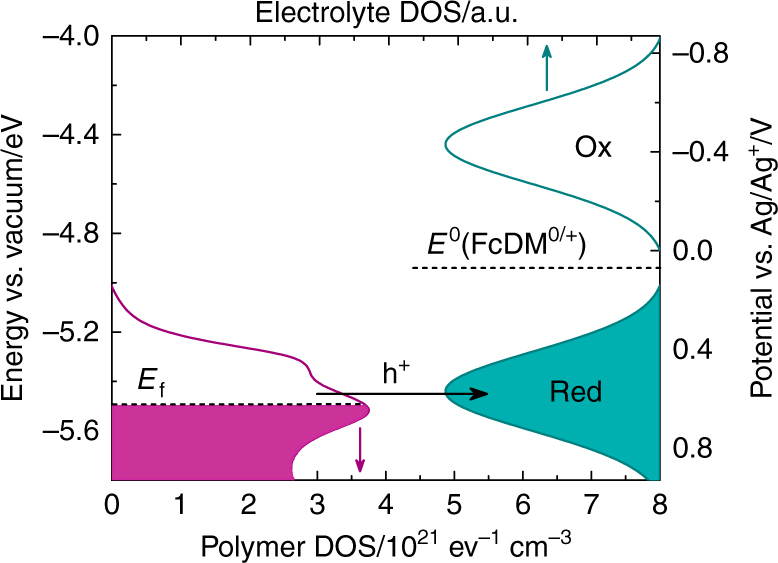
Distribution of unoccupied and occupied states in polymer and electrolyte. The DOS of an e-P3HT film derived from the experimental oxidation current in cyclic voltammetry (purple curve, bottom axis) is shown next to simulated distributions of occupied (Red) and unoccupied (Ox) states in the electrolyte according to Eq. (3) and an analogous expression for unoccupied states, assuming equal concentrations of red and ox (green curves, top axis). Simulation parameters were *E* ^0^(FcDM^0/+^) ≈ *E*_1/2_(FcDM^0/+^) = +0.07 V vs. Ag/Ag^+^, *λ* = 0.5 eV^50^, and *T* = 298 K. Occupancy of the P3HT DOS and hole transfer from polymer to the electrolyte are illustrated for the example of a Fermi level *E*_f_ at −5.5 eV at the surface of the polymer electrode

The effective DOS obtained for e-P3HT is described as a superposition of two Gaussian distributions (Fig. 2). The shoulder (~0.4 V) corresponds to more ordered domains, which are oxidized more easily than the amorphous phase (~0.65 V)^25, 36^. The calculated distributions of states are in good agreement with DOS measurements on spin-cast P3HT by means of potential-dependent electrochemical impedance spectroscopy (EIS)^37, 38^, and reflect similarities with the DOS observed using ultraviolet photoemission spectroscopy^26, 27^. For potentials positive of ~0.85 V vs. Ag/Ag^+^, the calculation according to Eq. (2) becomes more ambiguous as the anodic current could also include onset of side reactions, such as oxidation of small amounts of water in the organic solvent.

Coupling between the polymer DOS and electronic states in the electrolyte can be assessed by approximating the distribution function of the fluctuating energy levels in the electrolyte as^32^

3 $$\mathrm{DOS}^{\mathrm{red}} = {c^{\mathrm{red}}} \cdot \frac{1}{{\sqrt {4\lambda kT} }}exp\left( { - \frac{{{{\left( {E - e{E^0} - \lambda } \right)}^2}}}{{4\lambda kT}}} \right)$$

with the parameters as defined above; an analogous mathematical construct can be used to describe the unoccupied states of the oxidized FcDM^+^. A simulation according to Eq. (3) is shown on the right side of Fig. 2 on the same energy scale as the experimentally determined DOS of the polymer. From Fig. 2, the polymer DOS near *E* ^0^ of FcDM^0/+^ shows nearly no overlap with the density of oxidized electronic states (DOS^ox^) in the electrolyte (FcDM^+^), but good energetic overlap with the DOS^red^ of reduced electronic states (FcDM^0^). This result qualitatively explains why reduction of FcDM^+^ by e-P3HT is hindered at any given potential applied to the polymer electrode, as observed in Fig. 1c.

### Measurement and simulation of charge-transfer kinetics

For a quantitative analysis of the charge-transfer kinetics, EIS was used to measure the charge-transfer resistance *R*_ct_ for the system e-P3HT/FcDM as a function of potential and corresponding Fermi level *E*_f_. *R*_ct_ (in units of Ω) is generally defined as:

4 $${R_{{\rm{ct}}}}\left( {{E_{\rm{f}}}} \right) = \frac{1}{A}{\left( {\frac{{\partial J\left( {{E_{\rm{f}}}} \right)}}{{\partial V}}} \right)^{ - 1}}.$$

We note that *R*_ct_ inversely correlates with the rate constant^32^. Inserting Eq. (1) into Eq. (4) and integrating under consideration of the zero temperature limit of the Fermi-Dirac distribution yields the Marcus–Gerischer expression for *R*_ct_(*E*_f_):

5 $${R_{{\rm{ct}}}}\left( {{E_{\rm{f}}}} \right) = {\left[ {eA{k^{\rm{t}}}{c^{{\rm{red}}}} \cdot \mathrm{DOS}\left( {{E_{\rm{f}}}} \right) \cdot exp\left( { - \frac{{{{\left( {{E_{\rm{f}}} - e{E^0} - \lambda } \right)}^2}}}{{4\lambda kT}}} \right)} \right]^{ - 1}}.$$

Thus, we anticipate a voltage-dependent change in the resistance to charge transfer, which is expected to vary with electronic overlap in the effective DOS of the polymer and the FcDM.

**Fig. 3:**
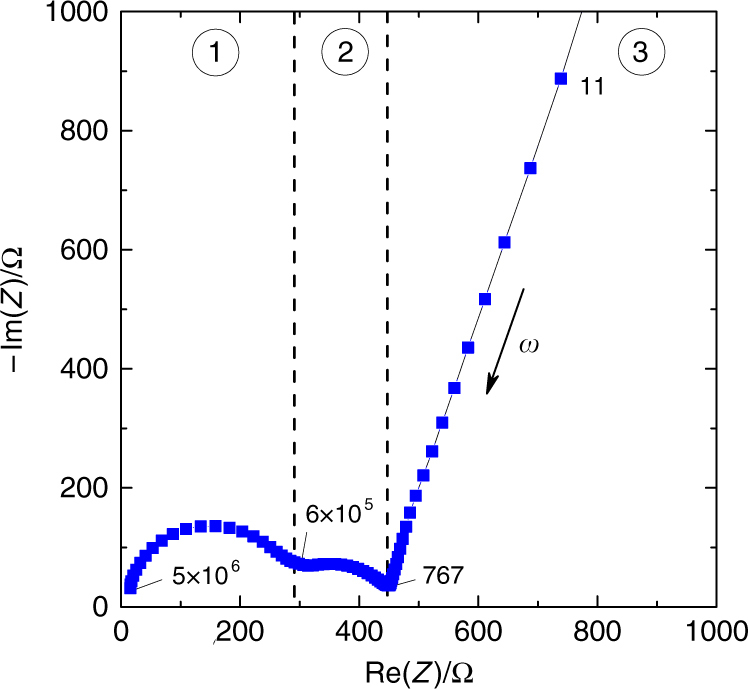
Typical Nyquist plot of the impedance of e-P3HT in 1 mM FcDM solution at a bias voltage of 0.35 V. Labels indicate the angular frequencies *ω* at select points

EIS spectra of the e-P3HT films were measured using freshly deposited samples that were conditioned by repeated cyclic voltammetry and pre-polarized at the given bias potential for 300 s in order to equilibrate the degree of doping and swelling of the polymer. An example is presented in Fig. 3. A full data set for the potential-dependent EIS Nyquist plots and corresponding Bode plots is provided in Supplementary Fig. 2, along with a detailed explanation of the spectral feature assignments (Supplementary Note 3). In Fig. 3, the Nyquist plot of the impedance of e-P3HT in contact with 1 mM FcDM solution at an intermediate applied potential demonstrates three resolvable features: a linear part at low angular frequencies (*ω*) (region 3 in Fig. 3) and two semicircles at intermediate and high frequencies (regions 2 and 1 in Fig. 3). Semicircles in EIS Nyquist plots are indicative of interfacial phenomena. The high-frequency feature has previously been ascribed to charge transfer and accumulation at the interface between substrate (in this case ITO) and polymer^39^. As expected from this assignment, both the resistance and the capacitance associated with this high-frequency semicircle showed little variation as a function of potential from 0.0 to 1.0 V (vs. Ag/Ag^+^) (see Supplementary Fig. 3). In contrast, the mid-frequency semicircle showed a significant change in diameter with the applied potential (cf. Supplementary Fig. 2), and is attributed to the combined impedance arising from charge transfer and double-layer capacitance at the P3HT/electrolyte interface.

**Fig. 4:**
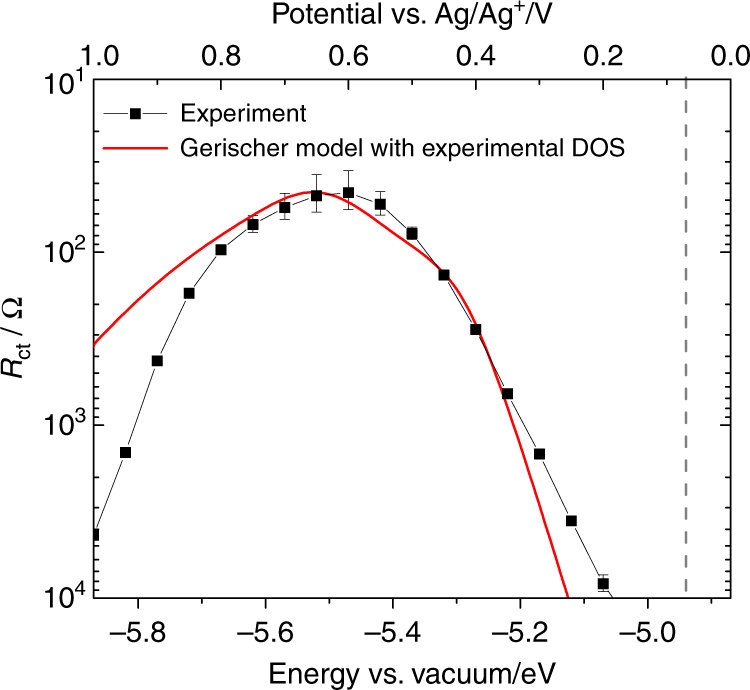
Charge-transfer resistance of e-P3HT in contact with ferrocenedimethanol solution. Experimental potential-dependent charge-transfer resistance obtained from fits of the impedance spectra (black symbols and line, with error bars indicating the fitting uncertainty), and simulation based on the Marcus–Gerischer model using the experimental DOS from cyclic voltammetry (red line). Simulation parameters (origins described in main text): *A* = 0.4 cm^2^, *k* ^t^ = 1.6 × 10^−22^ cm^4^ s^−1^, *c* ^red^ = 6 × 10^17^ cm^−3^, *λ* = 0.6 eV, *E* ^0^(FcDM^0/+^) = +0.07 V vs. Ag/Ag^+^ or −4.94 eV vs. vacuum (indicated by dashed gray line), *T* = 298 K

From the general assignment of spectral features, we fit the data with a modified Randles circuit including an additional, parallel *RC* element (cf. Supplementary Fig. 4) and extracted the potential-dependent resistances to charge transfer (*R*_ct_) at the polymer/electrolyte interface, see Fig. 4. The *R*_ct_ axis in Fig. 4 is inverted to reflect the inverse relationship between charge-transfer resistance and the rate constant of charge transfer given in Eq. (4). Analysis was repeated for nine polymer films, all of which yielded similar results to those presented in Fig. 4. EIS measurements in background electrolyte (without FcDM) resulted in similar results as the measurements with the redox species (cf. Supplementary Fig. 5), with the main difference being the diameter of the polymer/electrolyte charge transfer feature due to the absence of electronic charge transfer.

The shape of the curve in Fig. 4 is reminiscent of the normal and inverted regimes of the classic Marcus theory predictions for the fundamental rate constant *k*_et_ as a function of reaction Gibbs free energy (Δ*G*)^40^. The measured *R*_ct_ shows a general decrease from ~10^4^ Ω at +0.25 V to a local minimum of ~50 Ω at +0.6 V, followed by an increase to ~10^4^ Ω with increasing potential, from right to left in Fig. 4. As *R*_ct_ is inversely proportional to the charge-transfer rate constant, the minimum of the potential-dependent *R*_ct_ coincides with a local maximum in the rate constant near 0.6 V. For the present materials system, the inversion in charge-transfer kinetics is due to a decrease in DOS in the polymer at potentials positive of ~0.63 vs. Ag/Ag^+^ (local maximum in Fig. 2, left). Such behavior is distinct from charge transfer at ideal inorganic semiconductor/electrolyte junctions, which undergo band bending. In a band bending scenario, charge transfer predominantly occurs from a fixed energy level (conduction band edge or valence band edge) independent of the applied electrode potential^32, 41^. Specifically, for conventional inorganic materials, the charge-transfer rate is expected to exponentially increase with applied potential due to an exponential increase in the charge density in the space charge region near the surface (see Supplementary Note 4 and Supplementary Fig. 6).

A simulation of *R*_ct_(*E*_f_) according to the Marcus–Gerischer model (Eq. (5)) with our experimental density of states DOS(*E*_f_) of Fig. 2 is shown in Fig. 4 (red curve). The simulated curve adequately describes the potential-dependence of the experimental charge-transfer resistance up to a bias voltage of about 0.75 eV. Beyond that, the calculation becomes inaccurate because of ambiguity associated with the experimental values of DOS(*E*_f_), as described above. The model predicts that the low-potential shoulder in the DOS leads to a corresponding feature in the charge-transfer resistance (~0.4 V), but this was not observed experimentally due to the resolution limit of the EIS approach.

Alignment of the simulated and experimental *R*_ct_ in the minimum was achieved by setting the value of the time constant *k* ^t^ to 1.6 × 10^−22^ cm^4^ s^−1^, significantly smaller than the values reported for inorganic semiconductor/electrolyte junctions (10^−16^ to 10^−17^ cm^4^ s^−1^)^41–43^. For inorganic semiconductors, *k* ^t^ is associated with the electronic tunneling event across the interface and is distance-dependent. It is improbable that a difference in electronic tunneling probability is responsible for such a large difference in *k* ^t^ in this case. In the hypothetical case of only electronic charge transfer, then the probability of electron or hole tunneling is determined by the square of the coupling matrix element for initial and final states of the electron transfer reaction, ${\left| H \right|^2}$ ^32, 40, 41, 44, 45^. The latter depends primarily on the separation distance *R* between electrode and redox species in solution according to the exponential relationship:

6 $${\left| H \right|^2} = {\left| {{H_0}} \right|^2}\cdot {{exp}}\left( { - {\alpha _{\rm{R}}}\cdot R} \right),$$

where *α*_R_ is a function of the height of the potential barrier between electrode and redox species and is affected by the nature of the electronic coupling bridge^40, 44^. A decrease in tunneling probability by 10^5^ requires a larger *α*_R_·*R* term by 10^2^ for e-P3HT electrodes compared to inorganic semiconductors. *R* for inorganic semiconductors has been reported to be on the order of 5–10 angstroms^32, 46, 47^, which is comparable to the length of the hexyl side chain of P3HT (see Supplementary Fig. 8). Estimations of *α*_R_ for a variety of different electron transfer systems are all between 10 and 15 nm^−1^ (see ref. ^40^ and references therein), suggesting *α*_R_ is also very unlikely to change by 10^2^ with electrode material. Therefore, the value of *k* ^t^ in the case of a polymeric electrode is controlled by additional factors other than the tunneling probability of electronic charge carriers, and hence constitutes an effective kinetic parameter rather than a true tunneling time constant. Specifically, we suggest that *k* ^t^ reflects the influence of ion intercalation on the observed charge-transfer kinetics.

## Discussion

Combined electronic/ionic charge transfer is unique from the mechanisms considered by the original Marcus–Gerischer model, which was derived for purely electronic charge-transfer reactions at electrodes. For polymers, *k* ^t^ is certainly a more useful figure of merit for comparison of different materials systems than a standard rate constant *k* ^0^ from the Butler–Volmer approximation. Together with the DOS distributions of polymer and electrolyte, *k* ^t^ could therefore serve as a critical design parameter in the development of new materials systems for (photo-)electrochemical cells and other polymer-based electrochemical devices. One major challenge for organics is the relatively low value of *k* ^t^, which appears to depend both on the electronic coupling as well as on ion transport within the polymer. We suggest that molecular design should focus on controlling the overlap of the DOS of the polymer and the redox probe and optimizing the potential-dependent physical structure of the film to facilitate ion movement. If *k* ^t^ can be increased, the advantage of kinetic and thermodynamic selectivity could be leveraged for new devices and applications.

**Fig. 5:**
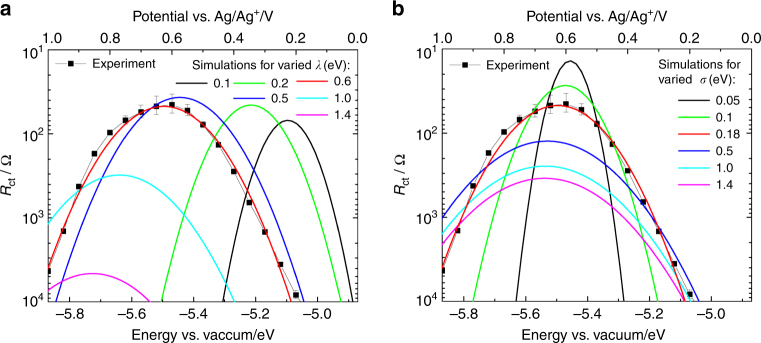
Effect of the distributions of electronic states in electrolyte and polymer on the charge-transfer resistance according to the Marcus–Gerischer model. **a** Experimental charge-transfer resistance (black symbols, with error bars showing the fitting uncertainty) and simulated charge-transfer resistances (colored lines) for varied reorganization energy *λ* of the electrolyte at fixed width *σ* = 0.18 eV of the Gaussian polymer DOS. **b** Experimental (black symbols with fitting uncertainty as error bars) and simulated (colored lines) *R*_ct_ for varied *σ* at fixed *λ* of 0.6 eV. Other simulation parameters were *N* = 10^21^ cm^−3^ (number of states per unit volume), *E*_ct_ = −5.45 eV (center of the Gaussian distribution), *A* = 0.4 cm^2^, *k* ^t^ = 1.6 × 10^−22^ cm^4^ s^−1^, *c* ^red^ = 6 × 10^17^ cm^−3^, *E* ^0^(FcDM^0/+^) = +0.07 V vs. Ag/Ag^+^ (or −4.94 eV), and *T* = 298 K. The red curves correspond to a simulation using the reorganization energy of the best fit shown in Fig. 4 (*λ* = 0.6 eV) together with the Gaussian DOS width that best describes the experimental DOS of the polymer (*σ* = 0.18 eV)

As a step toward creating polymer electrodes with kinetic or thermodynamic selectivity for charge transfer to certain redox probes, we consider how the various factors associated with electronic states distributions in polymer and electrolyte can influence charge-transfer kinetics for a given *k* ^t^. In Fig. 5, we consider how changes on either side of the interface alter the *R*_ct_, achieved by altering the reorganization energy *λ* of the redox probe (Fig. 5a) and width *σ* of the Gaussian DOS of the polymer (Fig. 5b).

Decreasing *λ* of the probe molecule results in a narrowing of the *R*_ct_ curve and a shift of the potential of minimum charge-transfer resistance (maximum charge-transfer rate constant) away from the maximum of the polymer DOS toward, and eventually beyond, the standard potential *E* ^0^ of the redox probe (here at −4.94 eV). Increasing *λ* requires larger over-potentials to achieve optimum charge-transfer kinetics, and large reorganization energies of 1 eV and more result in a substantial increase in the minimum value of *R*_ct_ by several orders of magnitude. Alternatively, in Fig. 5b, very narrow DOS distributions, such as those resulting from single polymer chains^48^, lead to narrower *R*_ct_ curves with a reduced resistance to charge transfer (increased rate constant). Increasing *σ* broadens the *R*_ct_ curve, shifts the minimum toward more negative energies (higher positive potentials), and increases the minimum value of the charge-transfer resistance assuming a constant tunneling constant *k* ^t^. These results are indicative of charge transfer being promoted by optimized charge (polaron and ion) transport pathways perpendicular to the electrode.

In conclusion, we demonstrate the kinetics of charge transfer at polymer/liquid interfaces are determined by the potential-dependent overlap in the density of unoccupied and occupied states in the polymer and the redox electrolyte. Most notable is the presence of the inverted charge-transfer regime at large over-potentials, a phenomenon not observed for inorganic semiconductor electrodes. The charge-transfer behavior at polymer electrodes is directly predicted by the Marcus–Gerischer theory, indicating a path forward involves feedback between synthesis and computation. Synthetic efforts should target control of: (i) the effective tunneling parameter *k* ^t^; (ii) the voltage-dependent DOS of the polymer; (iii) the overlap between polymer DOS and redox probe; and (iv) the relative rates of electron and ion mobility within polymer films. In particular, the presence of the inverted regime suggests a promising path forward to the use of multi-electron transfer redox species and/or a second redox species, which could open up a range of novel organic electrochemical opportunities and devices.

## Methods

### Materials and film formation

ITO-coated glass (Colorado Concept Coatings LLC, sheet resistance <15 Ω sq^−1^) was cut into 1″ by ≤1″ pieces and cleaned by successive sonication in Triton X-100 (Sigma, laboratory grade) diluted with DI water, a 1:1 (by volume) mixture of 2-propanol (Pharmco Aaper, 70%) and DI water, and pure 2-propanol. The ITO was etched for 8 s using hydriodic acid (Sigma-Aldrich, 57 wt.% in H_2_O, 99.99%), rinsed with DI water, and dried in a stream of nitrogen. The etched substrates were functionalized with 3-thiophene acetic acid (3-TAA) by immersion in a 10 mM solution of 3-TAA (Aldrich, 98%) in acetonitrile (Fisher Scientific, ≥99.9%) overnight. e-P3HT films were prepared on the cleaned and 3-TAA-functionalized ITO-coated glass substrates from a 10 mM solution of 3-hexylthiophene (Aldrich, ≥99%) in 0.1 M tetrabutylammonium hexafluorophosphate (TBAHFP; Accela Chembio Inc., ≥95%) in acetonitrile, resulting in a film thickness of ~20 nm^25^. Deposition was achieved by stepping the potential to +1.4 V for 0.5 s, followed by applying +1.35 V until a total charge of 5 mC had been transferred (ca. 15–20 s).

### Electrochemical measurements

A CH Instruments 920D potentiostat with a 3-electrode setup consisting of the ITO substrate as working electrode (active electrode area of 0.95 cm^2^), a gold counter electrode, and an organic Ag/Ag^+^ reference electrode containing 10 mM AgNO_3_ (BASi) and 0.1 M TBAHFP in acetonitrile was used. Assuming a standard potential *E* ^0^ of FcDM of +0.44 V vs. NHE, the potential of the Ag/Ag^+^ reference electrode was calibrated by cyclic voltammetry of FcDM^0^/FcDM^+^ at Pt (half-wave potential *E*_1/2_ = +0.07 V vs. Ag/Ag^+^ ≈ *E* ^0^) to correspond to +0.37 V vs. NHE. The electron energy with respect to vacuum was estimated based on the assumption that a potential of 0 V vs. NHE corresponds to an electron energy of −4.5 eV vs. vacuum level^49^, hence 0 V vs. Ag/Ag^+^ correspond to −4.87 eV vs. vacuum level. Cyclic voltammetry and EIS were performed immediately after electrodeposition using the CH Instruments 920D potentiostat with an open electrochemical cell (CH Instruments, active geometric electrode area *A* = 0.4 cm^2^). A Pt wire counter electrode and an organic Ag/Ag^+^ reference electrode were employed. The electrolyte consisted of 1 mM 1,1′-ferrocenedimethanol (FcDM; Aldrich, 98%) and 0.1 M TBAHFP in acetonitrile. The holding time at the cathodic starting potential of the cyclic voltammograms prior to starting the scans was 5 s. Before measuring the impedance spectrum at a given bias potential, the polymer was held at the bias potential for 5 min for equilibration of the doping level.

### Data availability

Data supporting the findings of this study are available within the article and its Supplementary Information file and from the corresponding author on reasonable request.

## Electronic supplementary material

Supplementary Information
