# Supplementary material for: Normal and inverted regimes of charge transfer controlled by density of states at polymer electrodes
Source: Nat Commun. 2017 Oct 19;8:1048. doi: 10.1038/s41467-017-01264-2 (PMC5715087; doi:10.1038/s41467-017-01264-2)
Supplement: Supplementary file 1 — Supplementary Information [file 41467_2017_1264_MOESM1_ESM.pdf]

Supplementary Note 1: Determination of effective DOS from cyclic voltammetry

We utilize the electronic current measured in cyclic voltammetry as our starting point to derive a "density of states". As discussed in previous work,[1,2] the current density in a voltammetry experiment is

$$J = -ed\frac{dn}{dt} = -ed\frac{dn}{d\mu}\cdot\frac{d\mu}{dt} = -ed\frac{dn}{d\mu}\cdot\frac{d(-eU)}{dt} = \upsilon de^2\frac{dn}{d\mu} = -\upsilon de^2\frac{dp}{d\mu} \quad (1)$$

where *e* is the elementary charge, *d* is the film thickness, *n* is the concentration of electrons, *μ* is the chemical potential, *U* is the applied potential, υ is the potential scan rate, *p* is the concentration of holes (in the form of polarons and bipolarons) and $\frac{dn}{d\mu} = -\frac{dp}{d\mu}$ because $n+p=N_\pi$=const (N_π being the density of π orbitals).

The chemical capacitance per unit volume, also referred to as redox capacity, redox capacitance, or pseudocapacitance, is defined as[1-3]

$$C_\mu = e^2\frac{dn}{d\mu} = -e^2\frac{dp}{d\mu} \quad (2)$$

so that

$$C_\mu\left[\frac{F}{cm^3}\right] = \frac{J}{\upsilon d} \qquad or \qquad C_\mu[F] = \frac{J}{\upsilon}A \quad (3)$$

where *A* is the geometric film area.

In electrochemical doping specific to our system, the presence of excess holes resulting from oxidization must be accompanied by anions to maintain electroneutrality. In other words, an observed change in the chemical potential *μ* with hole concentration *p* (and hence the chemical capacitance) is also due to ion intercalation. Because the polaronic signature in conductive polymers is easily tracked in spectroelectrochemistry, as opposed to the ion concentration, convention has followed that of classic semiconductors and capacitance is defined by the hole concentration. This has been accounted for in the literature by describing *μ* as the sum of an electronic and an ionic contribution and/or via electron-ion interaction terms.[1,2,4]

Based on the chemical capacitance calculated from the voltammetric current, we estimated the density of states, DOS, using the approximation (valid for doping levels > 10%)[5]

$$C_\mu = -e^2\frac{dp}{d\mu} = e^2 DOS(-\mu) \quad (4)$$

The thus-obtained DOS should indeed not be understood as a strictly electronic density of states comparable to the DOS in the solid state, but rather as an effective DOS that includes the influence of ion intercalation.

Supplementary Note 2: Estimation of k^0 from cyclic voltammetry using Nicholson's method

Nicholson[6] has numerically derived and tabulated a relationship between the separation of anodic and cathodic potentials (ΔE_p) in cyclic voltammetry and the factor

$$\psi = \left(\frac{D_O}{D_R}\right)^{\alpha/2}\cdot\frac{k^0}{\sqrt{\pi\cdot D_O\cdot\frac{nF}{RT}\cdot\sqrt{\nu}}} \quad (5)$$

where D_O and D_R are the diffusion coefficients of the oxidized and reduced redox species, *α* is the transfer coefficient, k^0 is the standard rate constant, *n* is the number of electrons transferred

in the oxidation/reduction reaction, F is the Faraday constant, R is the gas constant, T is the temperature, and v is the potential scan rate used in the cyclic voltammetry measurement. Provided the scan rate and diffusion coefficients are known, this allows calculating k^0 from ΔE_p.

Electrodeposited P3HT

The experimental peak potential separation for the oxidation and reduction of FcDM at electrodeposited P3HT is approximately 275 mV (cf. Fig. 1c in the main paper). While Nicholson's model does not extend to such high values of ΔE_p, a rough approximation of the corresponding factor ψ was obtained by plotting Nicholson's tabulated $\psi(\Delta E_p)$ values, fitting with a x^{-1} function, and extrapolating to higher peak potential separations. This way, we obtained $\psi \approx 0.11$. Using eq. (5) with $D_O = 7 \cdot 10^{-6}$ cm^2 s^{-1},[7] $n = 1$, $T = 298$ K, $v = 0.05$ V s^{-1}, and assuming that $D_O = D_R$,[8] this results in a standard rate constant $\boldsymbol{k^0 \approx 7 \cdot 10^{-4}}$ **cm s^{-1}**.

Platinum disk electrode

Supplementary Figure 1 shows the cyclic voltammogram of 1 mM FcDM in 0.1 M TBAHFP in acetonitrile measured at a Pt disk electrode (diameter = 2 mm) at a scan rate of 0.05 V s^{-1}. The peak potential separation is 87 mV, which according to Nicholson corresponds to $\psi \approx 0.9$. The analysis was repeated at 11 additional scan rates between 10 mV/s and 10 V/s, yielding an average value of the standard rate constant of $\boldsymbol{k^0 = 6 \cdot 10^{-3}}$ **cm s**$^{-1}$ (+/- $0.9 \cdot 10^{-3}$ cm s^{-1}).

Supplementary Note 3: Discussion of assignment of spectral features in EIS spectra

Our construction of an equivalent circuit (Supplementary Figure 4) started from an approach routinely followed in the conductive polymer literature, which is based on the modified Randles circuit.[9,10] An additional parallel RC element was introduced to account for the experimental observation of two interfacial features (semicircles) in the Nyquist plot of the impedance (cf. Fig. 3 in the main text). This type of circuit is applicable to substrate/polymer/electrolyte systems both with and without a redox-active species in the electrolyte. Specifically, the addition of a redox couple causes a transition from purely ionic charge transfer to hybrid electronic-ionic charge transfer at the polymer/electrolyte interface accompanied by a change in magnitude and frequency of the observed interfacial resistance (R_{ct}). This is indicated by the reference measurement without ferrocenedimethanol (Supplementary Figure 5) and has previously been discussed in the literature.[10-12]

Discussion of the capacitive elements CPE_{ITO} and CPE_{dl}

The only capacitances directly visible in the equivalent circuit are the (electrostatic) double-layer capacitances at the ITO/polymer (C_{ITO}) interface and at the polymer/electrolyte (C_{dl}) interface. Both have been generalized into constant phase elements CPE_{dl} and CPE_{ITO} to account for non-ideal frequency dependence. The fits for these two elements are plotted in Supplemental Figure 3 as a function of applied potential. Briefly, the capacitive-like elements showed nearly no dependence on the applied potential, as expected for double layer capacitances. The magnitude, as well as the frequency range (see below), further confirm that the two elements are associated with interfacial capacitances. As expected, the C_{ITO} was found to be smaller than the C_{dl}, as it is possible that the polymer double layer capacitance is occurring over more than a few atoms of thickness (increase in effective surface area).

It is important to note that these two circuit elements are observed in the higher frequency regime of the Nyquist plot (regions 1 and 2 in Fig. 3 of the main paper) as semicircles resulting from parallel combination of CPE_{dl} and R_{ct} and of CPE_{ITO} and R_{ITO}, where R_{ct} and R_{ITO} are the charge-transfer resistances at the respective interfaces.[12,13] It was this higher frequency range, at which the potential modulation is too fast for ions to follow and interfacial processes dominate the impedance, that we were most interested in in our present work. On the other hand, coupled ionic-electronic charge transport in the polymer film as well as charging (doping and de-doping) of the polymer are known to dominate the low-frequency part of the impedance spectrum (region 3 in Fig. 3).[9,12,14]

Insights on chemical capacitance

The doping/de-doping of the polymer in particular is described by the chemical capacitance C_{μ}, which is a bulk property of the polymer and is therefore expected to scale volumetrically. To clarify the relationship between C_{μ} and our equivalent circuit in Supplementary Figure 4, it should first be pointed out that a more accurate description of the low-frequency behaviour of polymer electrodes has been obtained by others by modification of the Randles circuit with a more general diffusion element Z_{D} replacing the Warburg impedance W. This element Z_{D} is directly correlated to the chemical capacitance of the polymer:[14]

$$Z_{\mathrm{D}} = \frac{d^2}{D \cdot C_{\mu}} \cdot \left(\frac{\mathrm{i}\omega d^2}{D}\right)^{-1/2} \cdot \coth\left(\frac{\mathrm{i}\omega d^2}{D}\right)^{1/2} \quad (6)$$

where d is the film thickness, and D is the diffusion coefficient. At frequencies $\omega << D/d^2$, Z_{D} is dominated by C_{μ}, while at $\omega > D/d^2$, a limiting behaviour equivalent to that of a Warburg impedance is approached.

Since our present study was focused on the charge-transfer resistance dominant at much higher frequencies, we simplified the fitting procedure by limiting the fitting range to frequencies that excluded the capacitance-dominated ultralow-frequency regime, and used a simple Warburg element in place of the more complex diffusion element Z_{D}. Note that a non-ideal Warburg element[15] with flexible exponent in the impedance function (as opposed to a fixed exponent of 0.5) was required to account for non-ideal experimental slopes in the Nyquist plot of > 45°. Such non-ideality is not unusual and has been ascribed by Láng and Inzelt[16] to inhomogeneities in the film thickness perturbing both Warburg and capacitive regime. Moreover, the low-frequency impedance can be affected by slow mass transport of the redox couple to the polymer surface.[10]

Supplementary Note 4: Additional simulations of R_{ct} according to the Marcus-Gerischer model

If the charge transfer associated with oxidation of a redox mediator at an electrode occurs mainly around the energy of the valence band edge E_{vb}, as is the case in conventional inorganic p-type semiconductor/electrolyte junctions that show band bending, the Marcus-Gerischer current density of eq. (1) in the main paper simplifies to[17]

$$J = ek^{\mathrm{t}}c^{\mathrm{red}}\exp\left(\frac{-\left(E_{\mathrm{vb}}^{\mathrm{s}} - eE^0 - \lambda\right)^2}{4\lambda kT}\right) \cdot DOS(E_{\mathrm{vb}}^{\mathrm{s}}) \cdot F(E_{\mathrm{vb}}^{\mathrm{s}}, E_{\mathrm{f}}) \quad (7)$$

where $E_{\mathrm{vb}}^{\mathrm{s}}$ is the energy of the valence band edge at the surface and all other parameters are defined in the main paper. Eq. (7) can be written as[17]

$$J = ek^{\mathrm{t}} c^{\mathrm{red}} p_{\mathrm{s}} \exp\left(\frac{-\left(E_{\mathrm{vb}}^{\mathrm{s}} - eE^0 - \lambda\right)^2}{4\lambda kT}\right) \tag{8}$$

in which p_s is the surface hole density. Since the exponential term in eq. (8) is independent of the applied potential, variation of the potential in this case only modulates p_s through changing the band bending and, hence, the separation of E_{vb} and E_f at the surface. The dependence of p_s on the applied potential is determined by the Boltzmann distribution function

$$p_{\mathrm{s}} = p_0 exp\left(\frac{e\Delta\varphi_{\mathrm{sc}}}{kT}\right) \tag{9}$$

where p_0 is the bulk hole density, $\Delta\varphi_{sc}$ is the potential drop across the space-charge layer associated with band bending, and changes in applied potential are assumed to be equal to changes in $\Delta\varphi_{sc}$.[17] Inserting eq. (9) into eq. (8) and applying eq. (4) of the main paper yields a formula for R_{ct} that was used for a simulation, see green curve in Supplementary Figure 6. For comparison, Supplementary Figure 6 shows the experimental data and a simulation for a simple Gaussian DOS without band bending according to eq. (5) in the main paper with a standard Gaussian function inserted for $DOS(E_f)$.

<u>Supplementary Note 5: Relationship between standard rate constant k^0 and Marcus-Gerischer tunnelling constant k^t</u>

In terms of a simple, pseudo-first order rate law, eq. (1) in the main paper can be expressed as

$$J = ek(E_{\mathrm{f}})c^{\mathrm{red}} \tag{10}$$

Comparison of this with eq. (1) of the main paper yields an expression for the pseudo-first order rate constant:

$$k(E_{\mathrm{f}}) = k_{\mathrm{t}} \int_E DOS(E) \cdot \left(1 - F(E, E_{\mathrm{f}})\right) \cdot exp\left(\frac{-\left(E - eE^0 - \lambda\right)^2}{4\lambda kT}\right) dE \tag{11}$$

Applying the zero temperature approximation of the Fermi-Dirac function $F(E,E_f)$, we obtain:

$$k(E_{\mathrm{f}}) = k_{\mathrm{t}} \int_{E\mathrm{f}}^{\infty} DOS(E) \cdot exp\left(\frac{-\left(E - eE^0 - \lambda\right)^2}{4\lambda kT}\right) dE \tag{12}$$

The integral was numerically solved in OriginPro for a range of E_f values, using the experimental DOS, E^0 = +0.07 V vs. Ag/Ag^+, λ = 0.6 eV, and kT = 0.027 eV. Along with $k^t = 1.6 \cdot 10^{-22}$ cm^4 s^{-1} (obtained from the fit of the potential-dependent R_{ct}, see Fig. 4 in the main paper), this resulted in $k(E_f)$ as plotted in Supplementary Figure 7 (black curve). For small voltages, the k vs. E_f behaviour can be approximated by a Butler-Volmer type exponential relationship (cf. fit in Supplementary Figure 7, and corresponding equation and fitting parameters k^0 and A in the inset of the figure). This allows to extract a standard rate constant of **$k^0 = (5.4 +/- 0.1) \cdot 10^{-3}$ cm s^{-1}** and a symmetry coefficient $\alpha = A \cdot kT = 0.33$.

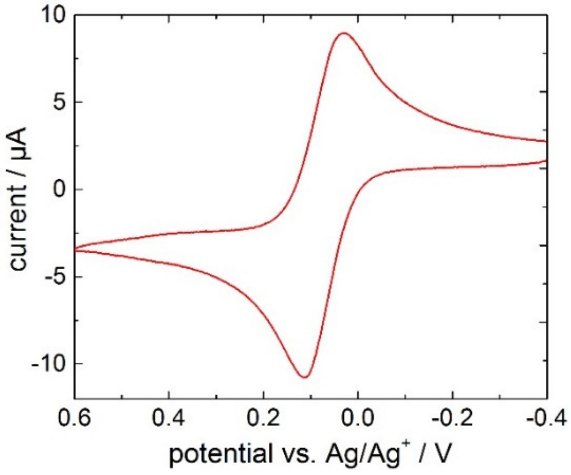

Supplementary Figure 1 | Cyclic voltammogram of 1 mM ferrocenedimethanol at Pt.

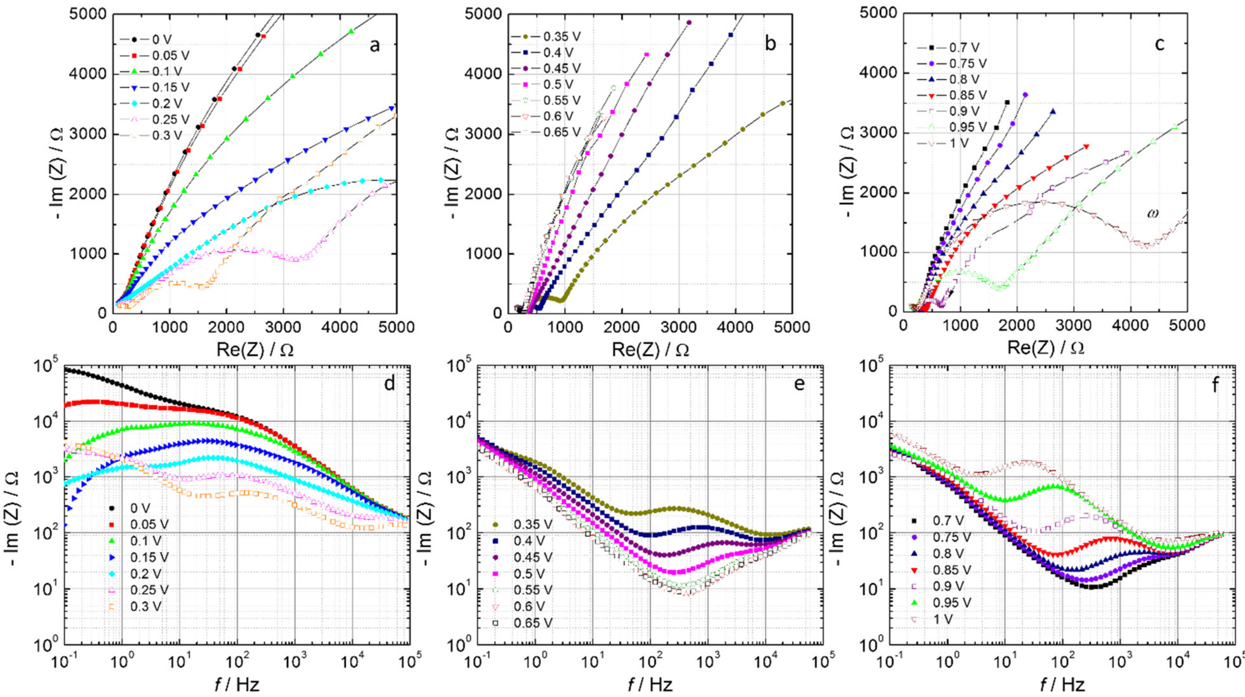

Supplementary Figure 2 | Potential-dependent impedance spectra of electrodeposited P3HT in contact with ferrocenedimethanol solution. (**a**)-(**c**) Nyquist plots for a series of applied potentials between 0 V and 1.0 V vs. Ag/Ag^{+}. (**d**)-(**f**) Bode plots of the imaginary part of the impedance for the same applied potentials.

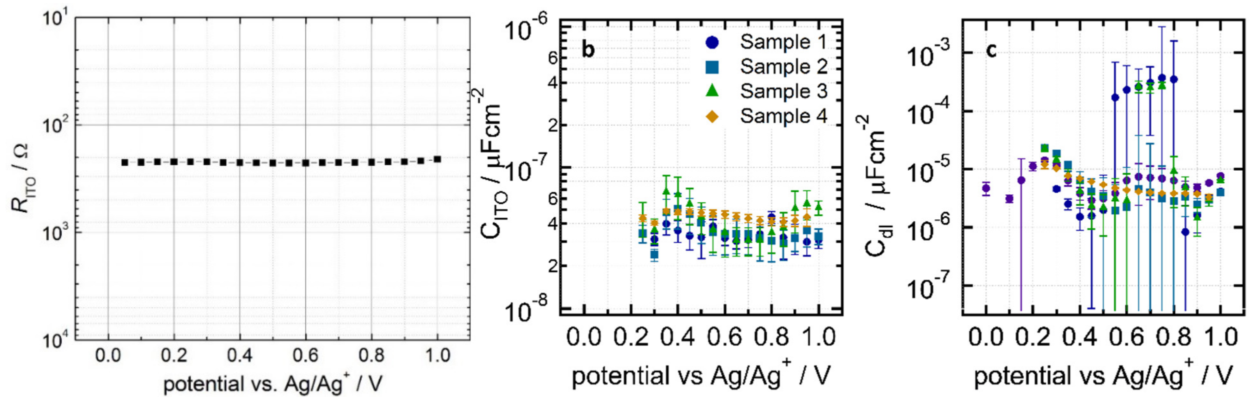

Supplementary Figure 3 | Potential-dependent resistances and capacitances obtained from fits of the impedance spectra. (**a**) Resistance of the interface between ITO and e-P3HT, $R_{ITO.}$ (**b**)-(**c**) Values of the pre-factor in the constant phase elements (*CPE*) associated with the non-ideal capacitances at the ITO/e-P3HT interface (C_{ITO}) and at the e-P3HT/electrolyte interface (C_{dl}). Error bars in (b) and (c) correspond to the fitting uncertainty.

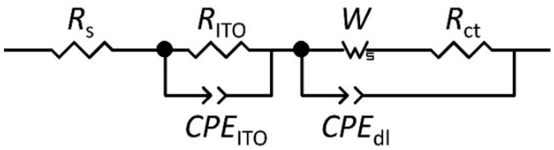

Supplementary Figure 4 | Modified Randles circuit used to fit the electrochemical impedance spectra. R_s is the series resistance, R_{ITO} and CPE_{ITO} are the resistance and constant-phase element associated with charge transfer and accumulation at the ITO/polymer interface, W is the Warburg impedance, CPE_{dl} is the double layer capacitance (constant-phase element) of the polymer/electrolyte interface, and R_{ct} is the charge-transfer resistance that reflects charge transfer at the polymer/electrolyte interface.

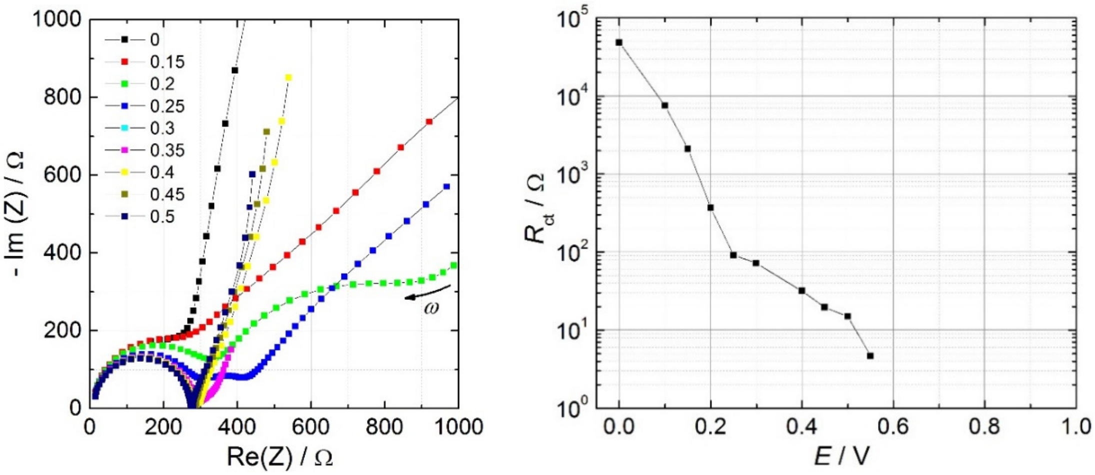

Supplementary Figure 5 | Potential-dependent impedance spectroscopy of electrodeposited P3HT in background electrolyte. (**a**) Nyquist plots of the impedance of e-P3HT in 0.1 M TBAHFP in acetonitrile. (**b**) Potential-dependent charge-transfer resistance obtained from fits of the spectra in (a).

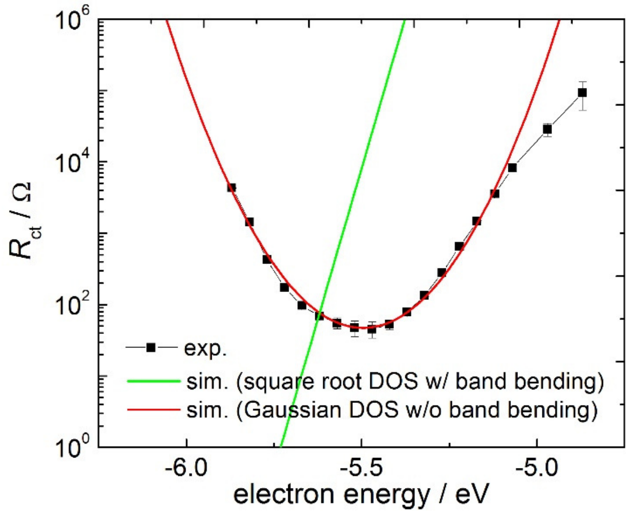

Supplementary Figure 6 | Experimental values and simulations of the energy-dependent charge transfer resistance. Experimental results of R_{ct} of an electrodeposited P3HT film in contact with ferrocenedimethanol solution (error bars show the fitting uncertainty) are compared to two simulations according to the Marcus-Gerischer model: (i) for a simple Gaussian *DOS* without band bending as observed in polymeric semiconductors (—), and (ii) for a square root-type DOS with band bending as characteristic for conventional inorganic semiconductors (—).

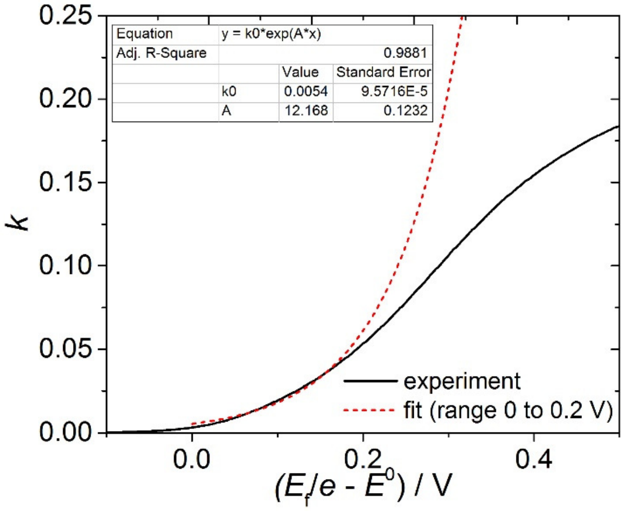

Supplementary Figure 7 | Estimation of a standard rate constant k^0 corresponding to the Marcus-Gerischer kinetic parameter k^t. The voltage-dependent pseudo-first order rate constant for the charge transfer between e-P3HT and ferrocenedimethanol calculated from experimental parameters including k^t (—) was fitted using the Butler-Volmer model (- - -) to estimate k^0.

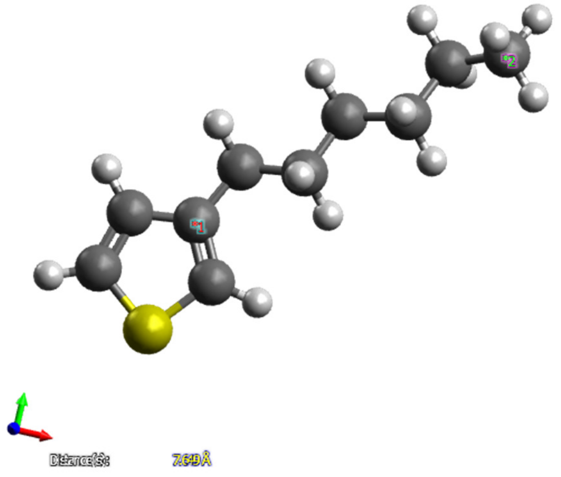

Supplementary Figure 8 | Molecular structure of 3-hexylthiophene. The structure of the monomer unit was drawn and measured using the software Avogadro, yielding a calculated length of the hexyl side chain of 7.69 angstroms (see bottom of figure).

Supplementary References

1 Bisquert, J., Garcia-Belmonte, G. & García-Cañadas, J. Effects of the Gaussian energy dispersion on the statistics of polarons and bipolarons in conducting polymers. *J. Chem. Phys.* **120,** 6726-6733 (2004)

2 Chidsey, C. E. D. & Murray, R. W. Redox capacity and direct-current electron conductivity in electroactive materials. *J. Phys. Chem.* **90**, 1479-1484 (1986).

3 Bisquert, J. Chemical capacitance of nanostructured semiconductors: its origin and significance for nanocomposite solar cells. *Phys. Chem. Chem. Phys.* **5**, 5360-5364 (2003).

4 Vorotyntsev, M. A. & Badiali, J. P. Short-range electron-ion interaction effects in charging the electroactive polymer-films. *Electrochim. Acta* **39**, 289-306 (1994).

5 Pomerantz, Z. *et al.* Capacitance, spectroelectrochemistry and conductivity of polarons and bipolarons in a polydicarbazole based conducting polymer. *J. Electroanal. Chem.* **614**, 49-60 (2008).

6 Nicholson, R. S. Theory and application of cyclic voltammetry for measurement of electrode reaction kinetics. *Anal. Chem.* **37,** 1351-1355 (1965).

7 Cannes, C., Kanoufi, F. & Bard, A. J. Cyclic voltammetry and scanning electrochemical microscopy of ferrocenemethanol at monolayer and bilayer-modified gold electrodes. *J. Electroanal. Chem.* **547**, 83-91 (2003).

8 Nioradze, N., Kim, J. & Amemiya, S. Quasi-Steady-State Voltammetry of Rapid Electron Transfer Reactions at the Macroscopic Substrate of the Scanning Electrochemical Microscope. *Anal. Chem.* **83**, 828-835 (2011).

9 Inzelt, G. & Láng, G. G. Electrochemical impedance spectroscopy (EIS) for polymer characterization. In *Electropolymerization*, Chapter 3, 51-76 (Wiley-VCH, 2010).

10 Rubinson, J. F. & Kayinamura, Y. P. Charge transport in conducting polymers: insights from impedance spectroscopy. *Chem. Soc. Rev.* **38**, 3339-3347 (2009).

11 Martini, M., Matencio, T., Alonso-Vante, N. & De Paoli, M. A. Electrochemical impedance spectroscopy of dodecylsulphate doped polypyrrole films in the dark and under illumination. *J. Brazil. Chem. Soc.* **11**, 50-58 (2000).

12 Garcia-Belmonte, G., Pomerantz, Z., Bisquert, J., Lellouche, J. P. & Zaban, A. Analysis of ion diffusion and charging in electronically conducting polydicarbazole films by impedance methods. *Electrochim. Acta* **49**, 3413-3417 (2004).

13 Vorotyntsev, M. A., Badiali, J. P. & Inzelt, G. Electrochemical impedance spectroscopy of thin films with two mobile charge carriers: effects of the interfacial charging. *J. Electroanal. Chem.* **472**, 7-19 (1999).

14 Rubinstein, I., Rishpon, J. & Gottesfeld, S. An AC-impedance study of electrochemical processes at Nafion-coated electrodes. *J. Electrochem. Soc.* **133**, 729-734 (1986).

15 Bisquert, J. & Compte, A. Theory of the electrochemical impedance of anomalous diffusion. *J. Electroanal. Chem.* **499**, 112-120 (2001).

16 Lang, G. & Inzelt, G. Some problems connected with impedance analysis of polymer film electrodes – effect of the film thickness and the thickness distribution. *Electrochim. Acta* **36**, 847-854 (1991).

17 Memming, R. *Semiconductor Electrochemistry* (Wiley-VCH, Weinheim, 2001).
